# Young ERC Masterclass Journal Club Article: The EVIDENCE Trial

**DOI:** 10.1016/j.resplu.2026.101518

**Published:** 2026-10-08

**Authors:** Abdulkarim A. Almazrua, Maria Mermiri, Philipp Meyer, Joyce Yeung

**Affiliations:** a Prince Sultan College for Emergency Medical Services, King Saud University, Riyadh, Saudi Arabia; b Anaesthesiology Department, University Hospital of Larissa, Larissa, Greece; c Düsseldorf Fire Brigade, Düsseldorf, Germany; d Warwick Clinical Trials Units, Warwick Medical School, University of Warwick, Coventry, the United Kingdom of Great Britain and Northern Ireland; e Critical Care Department, Heartlands Good Hope and Solihull Hospitals, University Hospitals Birmingham NHS Foundation Trust, Birmingham, the United Kingdom of Great Britain and Northern Ireland

**Figure:**
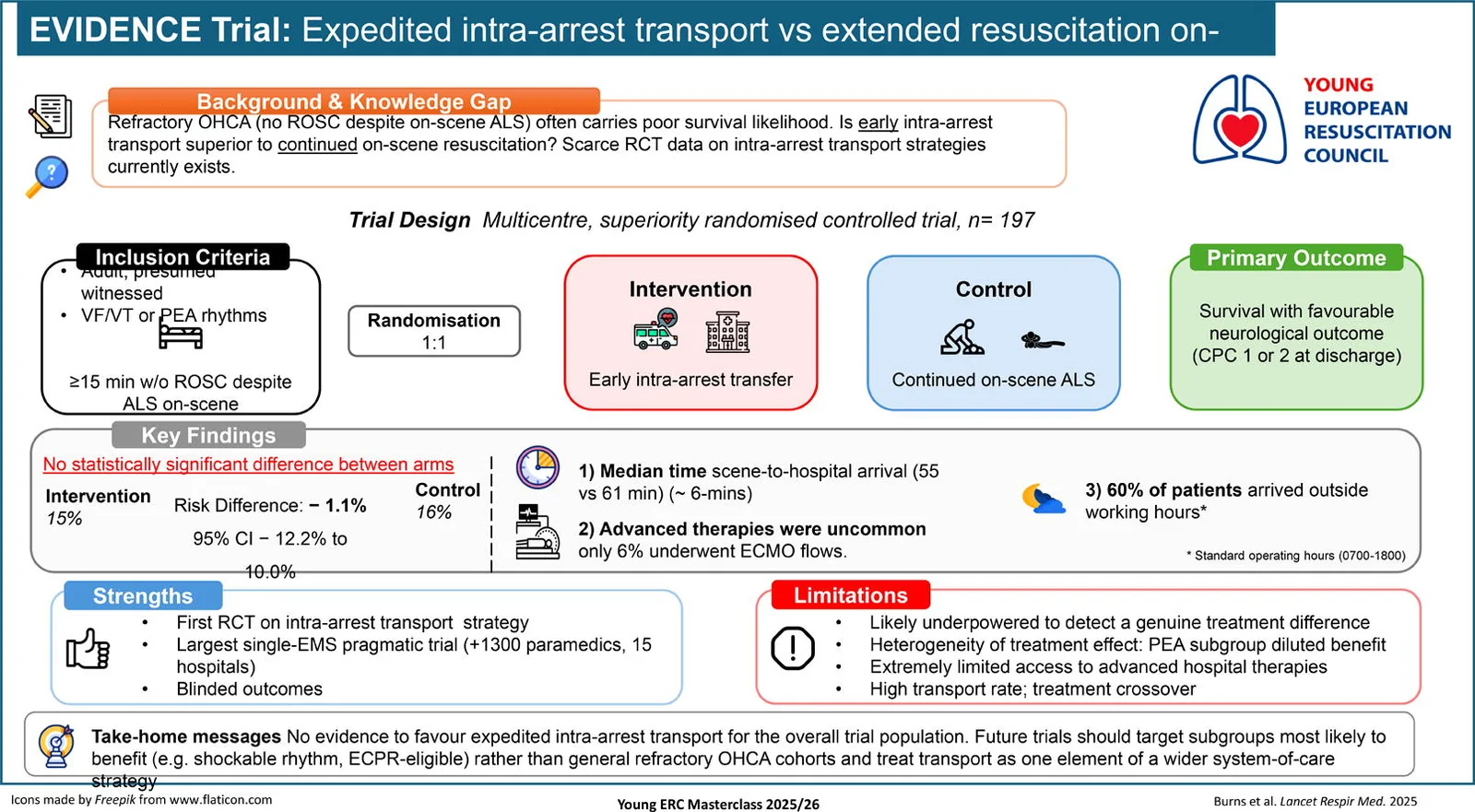

## Graphical illustration

This journal club article critically appraised the recent article “Expedited transfer from the scene for refractory out-of-hospital cardiac arrest in Australia: a prospective, multicentre, parallel, open-label, randomised clinical trial (EVIDENCE)”.^1^ A graphical abstract that summarises this trial can be found on the last page (Fig. 1). This work is part of the 2025/26 Young ERC Masterclass, the implementation of which has been discussed previously.^2^

**Fig. 1:**
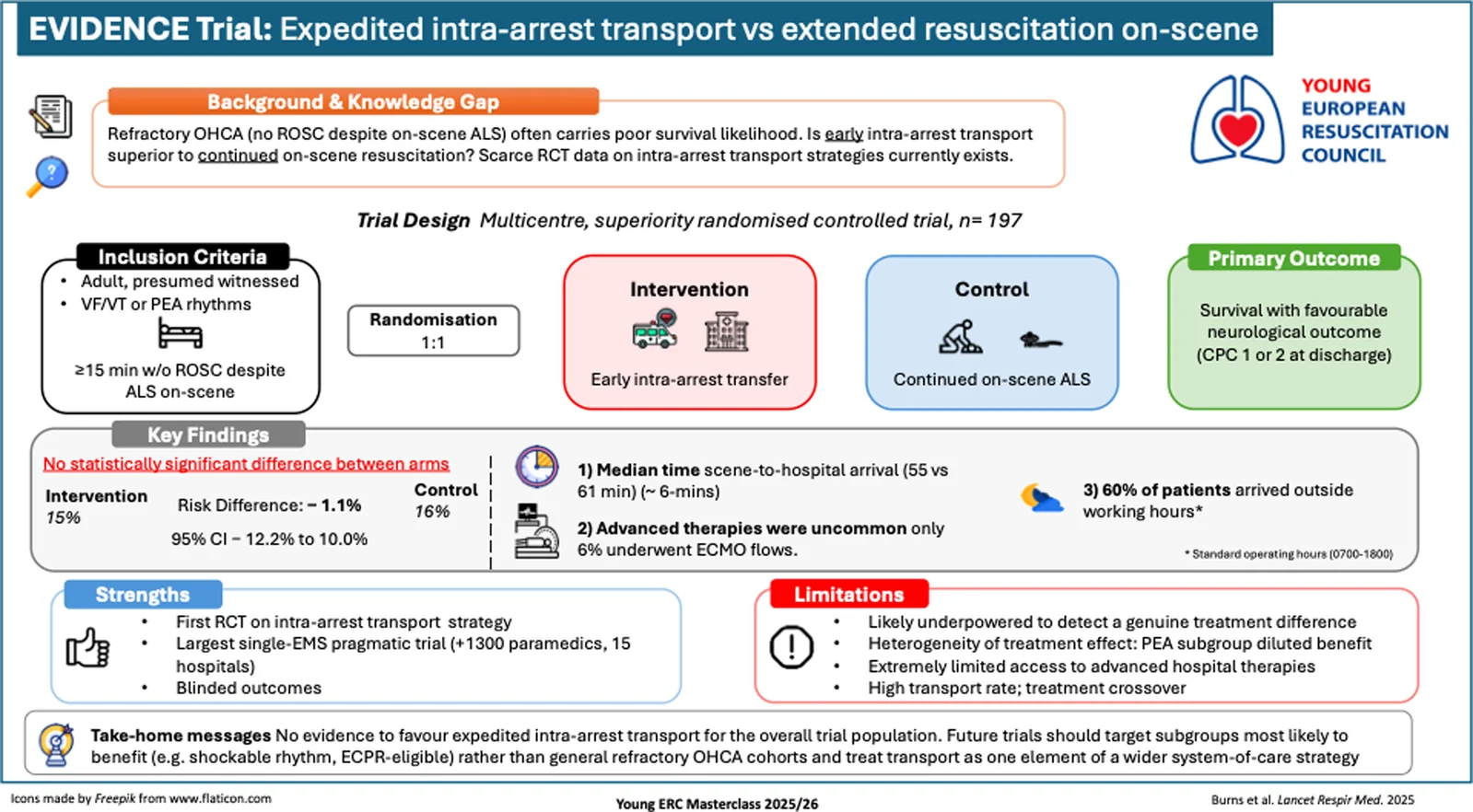

## Which knowledge gap does this study try to fill out?

Out-of-hospital cardiac arrest (OHCA) is associated with extremely poor survival, and outcomes deteriorate markedly when the arrest persists desipte standard resuscitation efforts. Observational studies report the likelihood of Return of Spontaneous Circulation (ROSC) worsening with longer durations of Cardiopulmonary Resuscitation (CPR), implying the ideal window for intra-arrest transport to hospital is within the first twenty minutes of initiating resuscitation.^4^, ^5^, ^6^

Whether expediting patient transport actually improves outcomes, however, remains unclear. Prior to the EVIDENCE trial, no randomised controlled trial had compared early intra-arrest transport against extended on-scene resuscitation in refractory OHCA. Existing evidence was limited to observational studies with inconsistent results, and recent trials either focused on Extracorporeal CPR (ECPR) as the primary intervention rather than transport strategy, or lacked a protocolised prehospital bundle.^3^, ^4^, ^5^, ^6^, ^7^, ^8^, ^9^, ^10^ EVIDENCE was designed to address this precisely.

## What was the design of the trial?

The EVIDENCE trial was a multicentre, parallel, prospective trial conducted across Greater Sydney, Australia (covering approximately 12,000 km^2^) including 15 receiving hospitals, five of which had ECPR capability. It is important to note that, in this region, Extracorporeal Membrane Oxygenation (ECMO) and other interventional cardiology teams were not accessible on-site outside standard hospital working hours (0700–1800).

Adults with witnessed OHCA of presumed medical origin and no prehospital ROSC after 15 min of advanced life support (ALS) were randomised 1:1 by paramedics. The intervention arm received an expedited transport bundle including mechanical cardiopulmonary resuscitation during extrication and transport; the control arm continued on-scene ALS. The primary outcome was survival with a favourable neurological outcome, defined as a Cerebral Performance Category (CPC) score of 1–2, at hospital discharge. A total of 197 refractory OHCAs were analysed.

## What were the key findings of the trial?

Favourable neurological outcome at hospital discharge occurred in 15% of the expedited group and 16% of the control group (risk difference −1.1%, 95% CI −12.2% to 10.0%). Advanced therapies were frequently inaccessible: only 6% underwent ECPR, approximately one-third of patients were transferred for coronary angiography, and of those, roughly half received percutaneous coronary intervention. The intervention achieved a six-minute reduction in arrest-to-hospital arrival (median time 55 vs. 61 min), yet 60% of patients arrived after working hours when advanced therapies were not offered.

## Are there any important methodological considerations to learn from the study?

A genuine methodological consideration demonstrated by this trial is the heterogeneity of treatment effect: the idea that a single average treatment effect estimated across a trial population may likely conceal meaningfully different effects within subgroups. In practice, when a trial enrols participants who are unlikely to benefit from the specific therapies the intervention is designed to facilitate, any true benefit among those most plausibly able to respond is very much diluted across the whole sample, risking a null result that reflects the composition of the enrolled patient population rather than a true absence of therapeutic benefit.^11^, ^12^

In the EVIDENCE trial, nearly one quarter of patients in each arm presented with Pulseless Electrical Activity (PEA), a cardiac rhythm associated with near-universally poor outcomes regardless of transport strategy. The trial confirms this directly: only one PEA patient survived across the whole cohort, compared with 29 of 149 patients presenting with a shockable cardiac rhythm. Enrolling a subgroup with such a prognosis, irrespective of allocation, dilutes the detectable signal among those most plausibly positioned to benefit from intervention. Broad inclusion criteria are a deliberate and defensible feature of pragmatic trial design, strengthening external validity and real-world generalisability, but this comes at a cost to internal precision. The remaining methodological challenges, including modest arm separation, an underpowered sample, and structural barriers to advanced therapy delivery, are addressed in the strengths and limitations section below.

## What are the most important strengths and limitations of the study?

This study carries a number of extremely important strengths. Most notably, it represents the first randomised trial intentionally designed to evaluate a protocolised prehospital transport strategy in refractory OHCA, rather than focusing solely on ECPR as the primary intervention. Its multicentre design, including 15 hospitals, a single ambulance service, and over 1300 paramedics across over 10,000 km^2^, enhances the generalisability of findings to real-world prehospital care systems. The pragmatic design reflects routine clinical practice and provides meaningful insight into the operational feasibility of expedited intra-arrest transport at scale. Additionally, neurological outcome assessment using CPC was conducted by blinded site investigators, reducing the risk of ascertainment bias for the primary endpoint.

Several limitations must nonetheless be acknowledged. The trial may have been underpowered to detect a smaller but clinically meaningful treatment effect, partly as a result of broad patient selection. The achieved reduction in hospital arrival time was modest at six minutes, and most patients arrived outside these hours, meaning the intervention could rarely be delivered as intended. This is likely a setting-specific limitation that may differ from the resources available at other provinces or institutions, limiting the generalisability of these findings to settings with wider out-of-hours access. Also, the trial was revised to assume 25% neurologically favourable survival in the expedited arm (compared to 10% in controls), yet the observed rate was 15%, meaning the actual between-arm difference was much smaller than the trial was powered to detect. Moreover, neurological outcomes were assessed using the CPC rather than the modified Rankin Scale (mRS), as recommended by the International Liaison Committee on Resuscitation Core Outcome Set for Cardiac Arrest (ILCOR COSCA) statement,^13^ which may limit comparability with studies adhering to standardised outcome reporting. Additionally, the relatively long transport times observed in this trial (median time from arrest to hospital arrival of 55 min in the expedited arm vs 61 min in the control arm) may limit the generalisability of these findings to urban settings characterised by much shorter prehospital transport times. Finally, intra-arrest transport occurred in both arms, narrowing the distinction between groups and representing an important source of treatment contamination that may have somewhat diluted the true effect.

In general, the EVIDENCE trial is a reminder that even the most carefully designed trial cannot fully anticipate the operational realities of delivering a time-sensitive intervention across a large, complex prehospital care system.

## How will the results affect your clinical practice?

This trial does not support a single, universal policy of expedited transport strategy for all refractory OHCAs. Transport decisions should be tailored, guided by on-scene feasibility, chances of ROSC with prolonged resuscitation, availability of alternative interventions such as dual sequential defibrillation, and genuine access to advanced therapies in hospitals. High-performance CPR and maximising the likelihood of ROSC before transport remain the key priorities.

## What do you see as the next steps in research?

Regarding future research, it is essential to determine whether the neutral results obtained reflect a genuine lack of benefit from expedited transport or, alternatively, a mismatch between the intervention and those specific patient cohorts most likely to benefit from time-sensitive hospital-based interventions. Priority should be given to trials targeting clearly defined subgroups with a plausible in-hospital target (e.g., shockable rhythm, suspected coronary occlusion, or early eligibility for ECPR). Furthermore, studies should move beyond transport as an isolated intervention and instead evaluate whole-system configurations. This includes optimising prehospital identification pathways, direct triage to cardiac arrest centres, and activation models that minimise arrest-to-cannulation or arrest-to-reperfusion times. Such approaches may enable the development of more refined transport criteria, reduce non-beneficial intra-arrest movement, and better align limited specialist resources with patients most likely to benefit.

## Ethical approval

Not required. This journal club is a commentary paper on a recently published trial data and involved no new human participants or identifiable patient data.

## Disclosure of AI tool use

The authors of this paper used the artificial intelligence tool Claude (Anthropic) to assist with structuring the manuscript and improving the clarity of wording. All AI-generated outputs were reviewed and verified by the researchers, who remain fully responsible for the accuracy of the final content of the research.

## CRediT authorship contribution statement

**Abdulkarim A. Almazrua:** Writing – review & editing, Writing – original draft, Visualization, Validation, Conceptualization. **Maria Mermiri:** Writing – review & editing, Writing – original draft, Visualization, Conceptualization. **Philipp Meyer:** Writing – review & editing, Writing – original draft, Conceptualization. **Joyce Yeung:** Writing – review & editing, Visualization, Supervision, Conceptualization.

## Funding

This work received no specific grant from any funding agency.

## Declaration of competing interest

The authors declare that they have no competing financial interests that could have influenced the work reported in this journal club. Three of the authors (AA, PM, and MM) are participants in the Young ERC Resuscitation Science Masterclass 2025/26.
